# Diagnostic accuracy and usability of the EMBalance decision support system for vestibular disorders in primary care: proof of concept randomised controlled study results

**DOI:** 10.1007/s00415-021-10829-7

**Published:** 2021-10-20

**Authors:** Doris-Eva Bamiou, Dimitris Kikidis, Thanos Bibas, Nehzat Koohi, Nora Macdonald, Christoph Maurer, Floris L. Wuyts, Berina Ihtijarevic, Laura Celis, Viviana Mucci, Leen Maes, Vincent Van Rompaey, Paul Van de Heyning, Irwin Nazareth, Themis P. Exarchos, Dimitrios Fotiadis, Dimitrios Koutsouris, Linda M. Luxon

**Affiliations:** 1 The Ear Institute, University College London, London, WC1X 8EE UK; 2 University College London Hospitals NHS Trust, London, UK; 3 NIHR University College London Hospitals Biomedical Research Centre, London, UK; 4 1st Department of Otorhinolaryngology, Head and Neck Surgery, National and Kapodistrian University of Athens, Hippocrateion General Hospital, Athens, Greece; 5 Clinic of Neurology and Neurophysiology, Medical Center, Faculty of Medicine, University of Freiburg, Freiburg, Germany; 6 Antwerp University Research Centre for Equilibrium and Aerospace, University of Antwerp, Antwerp, Belgium; 7 Laboratory for Equilibrium Investigations and Aerospace, University of Antwerp, Antwerp, Belgium; 8 Department Otorhinolaryngology-Head and Neck Surgery, Antwerp University Hospital, University of Antwerp, Antwerp, Belgium; 9 School of Science, Western Sydney University, Sydney, NSW Australia; 10 Department of Rehabilitation Sciences, University of Ghent, Ghent, Belgium; 11 Department of Primary Care and Population Health, University College London Medical School, London, UK; 12 Department of Informatics, Ionian University, Corfu, Greece; 13 Unit of Medical Technology and Intelligent Information Systems, Department of Materials Science and Engineering, University of Ioannina, Ioannina, Greece; 14 Biomedical Engineering Laboratory, National Technical University of Athens, Athens, Greece

**Keywords:** Dizziness, Diagnosis, Randomised control trial, Decision support system

## Abstract

**Background:**

Dizziness and imbalance are common symptoms that are often inadequately diagnosed or managed, due to a lack of dedicated specialists. Decision Support Systems (DSS) may support first-line physicians to diagnose and manage these patients based on personalised data.

**Aim:**

To examine the diagnostic accuracy and application of the EMBalance DSS for diagnosis and management of common vestibular disorders in primary care.

**Methods:**

Patients with persistent dizziness were recruited from primary care in Germany, Greece, Belgium and the UK and randomised to primary care clinicians assessing the patients with (+ DSS) versus assessment without (− DSS) the EMBalance DSS. Subsequently, specialists in neuro-otology/audiovestibular medicine performed clinical evaluation of each patient in a blinded way to provide the “gold standard” against which the + DSS, − DSS and the DSS as a standalone tool (i.e. without the final decision made by the clinician) were validated.

**Results:**

One hundred ninety-four participants (age range 25–85, mean = 57.7, SD = 16.7 years) were assigned to the + DSS (*N* = 100) and to the − DSS group (*N* = 94). The diagnosis suggested by the + DSS primary care physician agreed with the expert diagnosis in 54%, compared to 41.5% of cases in the − DSS group (odds ratio 1.35). Similar positive trends were observed for management and further referral in the + DSS vs. the − DSS group. The standalone DSS had better diagnostic and management accuracy than the + DSS group.

**Conclusion:**

There were trends for improved vestibular diagnosis and management when using the EMBalance DSS. The tool requires further development to improve its diagnostic accuracy, but holds promise for timely and effective diagnosis and management of dizzy patients in primary care.

**Trial registration number:**

NCT02704819 (clinicaltrials.gov).

## Background

Dizziness and imbalance are common symptoms with a high socioeconomic impact. They occur in up to 40% of the population by 60 years of age and are amongst the most common symptoms for visits to a doctor [1, 2]. Patients with vertigo attend up to 9.6 visits with primary care physicians and up to 7.2 visits with specialists, and report undergoing six laboratory based diagnostic procedures [3]. Eighty percent of affected adults require sick leave from work [4] and 48% report significant disruption in their social and family life, and the need to change or even give up work [5]. In addition, these individuals have a higher risk for both cognitive and psychological impairment [6, 7]. Despite the frequency and the potentially detrimental impact of these problems, an average of 4.5 visits with healthcare providers will be required for affected individuals to receive a correct diagnosis and appropriate treatment plan [8]. Non-specialist physicians can be overwhelmed when faced with a patient complaining of these symptoms, due to the vagueness of the symptom report, the plethora of underlying pathologies, complexity of balance control mechanisms, and the lack of medical expertise [9, 10], resulting in late diagnosis and mismanagement of patients with vestibular disorders [8]. The majority of acute vertigo cases that present to emergency departments will be “benign” and due to vestibular conditions like BPPV, Meniere’s disease, acute unilateral vestibulopathy and vestibular migraine, with stroke estimated to account for only 4–15% of these cases [11]. However, posterior fossa (brainstem and cerebellar) strokes have a mortality of 3–19% [12, 13] and require prompt diagnosis and management to prevent further deterioration and promote recovery [14]. Misdiagnosis of posterior fossa stroke is more likely when patients report dizziness [15]. A structured diagnostic approach has been proposed for the evaluation of vertigo in an acute setting, to establish time onset of symptoms and their evolution, symptom triggers and appropriate examination as per the TiTraTE algorithm [16]. A combination of three oculomotor signs known as the HINTS [Head Impulse Test, (Gaze-) Nystagmus, Test of Skew (deviation)] assessment identify posterior fossa strokes with greater sensitivity than early MRI-DWI (100% vs. 72%) [17]. These vestibular conditions place a significant burden on health services, health economics and society [10, 18].

Advances in computer science and artificial intelligence may help address this unmet need with the development of computer systems that support clinical diagnosis [19] and therapeutic and treatment decisions based on personalised patient data [20, 21]. Decision Support Systems (DSS) in particular, aim to codify and strategically manage biomedical knowledge to handle clinical challenges using computer modelling tools, medical data processing techniques and artificial intelligence methods [22–24]. The coronavirus pandemic has accelerated telehealth developments within the vestibular field. A recent taskforce of vestibular and eye movement experts for the remote assessment of the dizzy patient via different commercially available virtual platforms proposed a diagnostic and a triaging strategy for urgent or expedited face to face outpatient assessment according to signs and combination of symptoms and symptom characteristics [25]. The taskforce concluded that eye movement examination including nystagmus, saccades, smooth pursuit, test of binocular alignment and head thrust test could be supported by these virtual platforms. A further feasibility study evaluated whether smartphone-based video recordings of positional testing could help screening of nonacute benign paroxysmal positional vertigo, with promising results [26]. However, while this work has developed some initial rules, it has not as yet resulted into a new DSS.

There are very few DSS that have been developed to diagnose vestibular disorders up to now [27, 28]. There is also an ongoing clinical study with a cluster-randomised controlled trial with a parallel-group design within a primary care setting in Germany, that evaluates use of a system that incorporates a computerized clinical decision system, a mobile application, and a counselling and interdisciplinary educational programme developed by the German Centre for Vertigo and Balance Disorders (DSGZ) (Computerised clinical decision system and mobile application with expert support to optimize management of vertigo in primary care: study protocol for a pragmatic cluster-randomised controlled trial [29].With the exception of the study by the German group, which is ongoing, these have not yet been validated in a real clinical setting with a non-specialist physician obtaining clinical information with the system’s support towards formulation of a diagnosis.

In addition, the majority of previous DSSs mainly target diagnosis, but none provide specific management including rehabilitation support for patients with vestibular disorders.

The EMBalance project [27, 30] aimed to develop and validate a web-based platform used by primary care physicians for the early diagnostic evaluation, and effective management of balance disorders. Herein, we describe the proof of concept clinical evaluation of the EMBalance DSS by means of a study conducted as per the published protocol [31].

## Aims

The primary aim was to assess the diagnostic accuracy of primary care physicians with (+ DSS) and without (−DSS) using the DSS in patients presenting with symptoms of vestibular disorders. Diagnostic accuracy was measured by level of agreement between the non-specialist physicians’ overall final diagnosis against the “gold standard” diagnosis that was made by the specialist (primary outcome measure).

The secondary aims were to examine DSS useability:

1. By examining the primary care clinical diagnosis of the + DSS versus the − DSS group by means of overall diagnosis and individual disorder diagnosis sensitivity, specificity, positive and negative predictive values (odds ratio).
2. By examining the diagnostic accuracy of the DSS as a standalone tool.
3. By examining the level of agreement between the non-specialist and DSS overall management against the “gold standard” management by the specialist.
4. By examining the level of agreement between the DSS standalone tool management against the “gold standard” management by the specialist.
5. By comparing the number of referrals to secondary care for management in both + DSS and − DSS groups.

## Methods

### Study design and settings

This clinical study was a randomised controlled trial. The EMBalance study was carried out simultaneously in the United Kingdom, Germany, Greece and Belgium. Table 1 provides the list of both primary and tertiary care centres participating in this study.

**Table 1:** Participating clinical settings

Institution	Primary care setting	Tertiary care setting
Greece (UoA^1^)	Hippocrateio Hospital	Hippocrateio Hospital
Belgium (UA^2^)	Antwerp University Hospital	Antwerp University Hospital
Germany (UKFLR^3^)	Freiburg University Medical Centre	Freiburg University Medical Centre
UK (UCL^4^)	Keats Group Practice Hampstead Group Practice Parliament Hill Practice James Wigg Practice Ampthill Practice West Hampstead Medical Centre Brondesbury Medical Centre	National Hospital for Neurology and Neurosurgery Royal National Throat, Nose and Ear Hospital

^1^University of Athens

^2^University of Antwerp

^3^University of Freiburg

^4^University College London

Ethical approval was obtained from the Yorkshire and The Humber—Bradford Leeds Research Ethics Committee (approval No. 16/YH/0051). The trial was registered in clinicaltrials.gov (ref. number: NCT02704819). The EMBalance DSS was reviewed and approved by the Medicines and Healthcare products Regulatory Agency (MHRA), based on the fact that the EMBalance DSS is a diagnostic support tool that is not intended to be a substitute for the clinician’s decision-making capacity.

### Participants

Patients who presented in primary care with balance related symptoms were recruited according to the following inclusion and exclusion criteria**:**

#### Inclusion criteria

- Aged 18–90 years
- Competent to understand the information provided
- Acute onset vertigo (single or multiple attacks; vertigo defined as movement sensation/illusion; onset less than 1 month before study recruitment) or chronic dizziness (defined as a sensation of disturbed or impaired spatial orientation without a false or distorted sense of motion with a duration of more than 3 but less than 12 months before study recruitment) that is exacerbated by head movements
- Sub-acute presentation of vertigo or dizziness (defined as above, with duration 0 to 3 months before study recruitment) without presentation to emergency services

#### Exclusion criteria

- Participants with learning disability or dementia or uncontrolled psychiatric disorders
- Pregnant and breastfeeding women
- Patients incapable or unwilling to give informed consent

### Study groups

Consented patients were randomised to the following study groups:

- *Intervention group (non-specialist doctor using the DSS, + DSS)* patients evaluated by a non-specialist doctor with the support of the DSS.
- *Control group (non-specialist doctor not using the DSS, −DSS)* patients evaluated by a non-specialist doctor without the support of the DSS.

### The theoretical basis of the intervention

The overall concept of the EMBalance project [31] and the methodology for the EMBalance DSS development [27] have been previously described. Briefly, the participating medical partners first established and agreed “gold standard” criteria for the diagnosis of balance disorders and treatment guidelines, following nomenclature, classification and recommendations of the Bárány Society (http://www.baranysociety.nl/), i.e. an international neuro-otological society with the key aim of formulating worldwide evidence-based consensus and standardisation in the vestibular science and clinical practice. The clinical partners then collected extensive retrospective clinical data that included medical history, signs and symptoms, audio-vestibular tests, imaging studies and questionnaires on 984 patients with diagnosed vestibular disorders. These data populated a specially constructed repository that was constructed after an analysis of EMBalance targeted user requirements and usage scenarios. The repository stored patient personal data, clinical history (e.g. symptoms, examinations, etc.), different kinds of pre-existing diseases and medications, and data regarding diagnosis and treatment planning produced by the DSS reasoning engine. Data mining techniques were used to identify and extract all useful parameters for the development and training of the algorithms that were embedded in the DSS. These algorithms, in combination with indicative parameters provided by the clinical partners, pre-defined the set of decisions that can be formulated by the system on the basis of the patient’s clinical data [27]. These components were then integrated with the required user-friendly interfaces to provide the complete EMBalance DSS platform [31].

### The EMBalance DSS

**Fig. 1:**
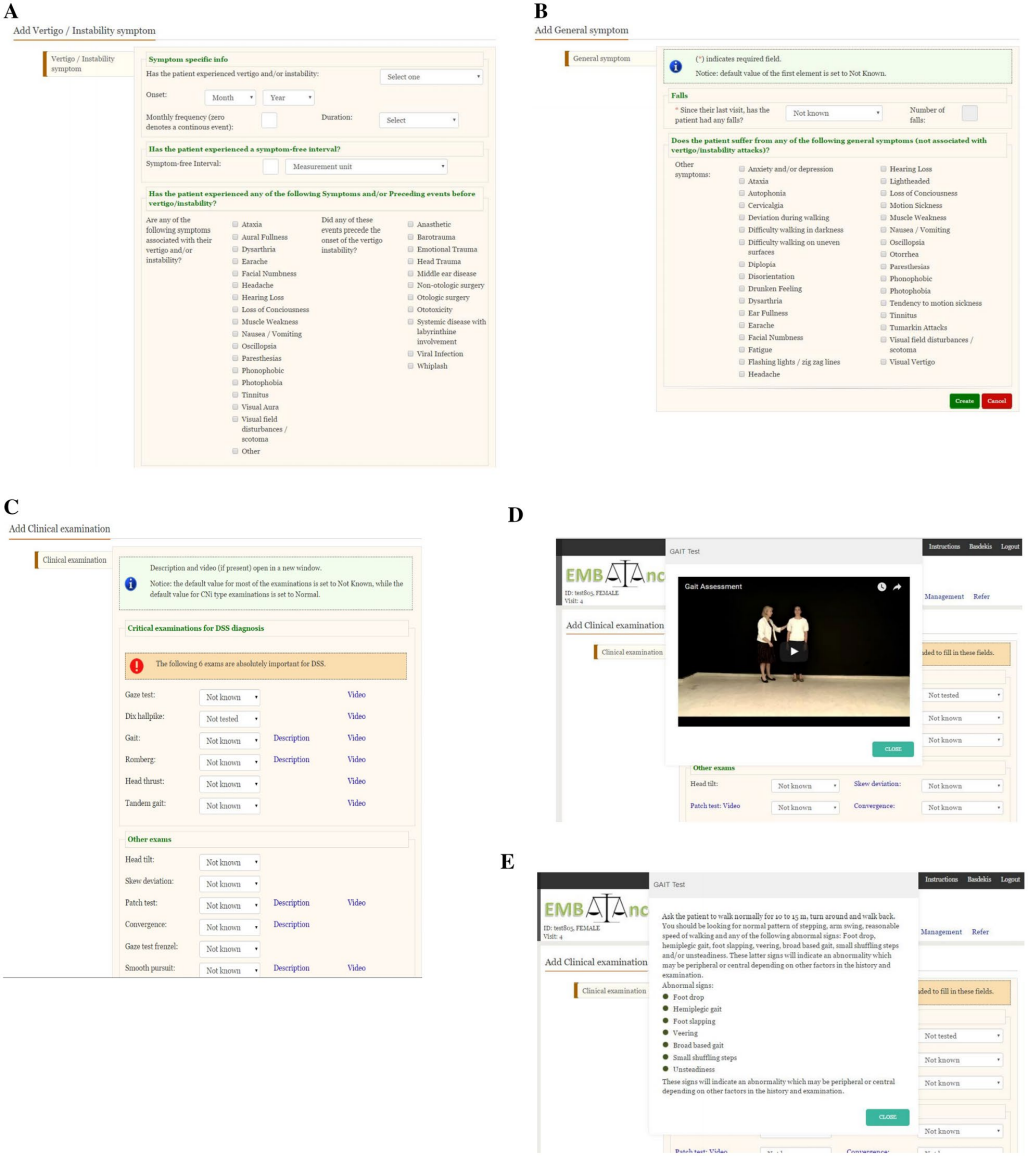
**A**–**E** provide some example screenshots for the history and examination taking process with the DSS. **A** DSS guided history taking—questions regarding vertigo and instability; **B** DSS guided history taking—questions regarding general symptoms; **C**, **D**, **E** Examples of DSS guided clinical examination with videos/instructions

The EMBalance DSS [27, 30] is a multi-language platform that consists of three modules:

- The database of the system, with the implementation of the repository that is composed of 48 entities, including instance tables (actual clinical data collected from 984 patients’ records), and type tables (e.g., patient occupation). The EMBalance repository characterises patients using approximately 350 features. These features include epidemiological characteristics, primary and secondary symptoms (defined as per the Bárány Society guidelines [32], symptom duration and frequency, existence and duration of symptom free intervals, accompanying symptoms (like disequilibrium, difficulty walking in uneven surfaces, motion sickness, headaches, disorientation, nausea etc), symptom triggers, comorbidities, clinical examination (that included HINTS) and audiological testing. These were not obligatory fields and users were free to populate as many fields as they thought relevant, however, the interface presented key aspects of the history/examination first to prompt the respondent to populate these fields. The interface was stable with no changes based on algorithmic rules, since the DSS used data mining techniques rather than algorithms. Several different data mining models were used with a different model developed for each disease, to allow for extraction of more than one diagnosis for each patient [27], as this is often the case for patients with balance disorders.
- The back end implements the functionalities of the system.
- The graphical user interface, also known as “*front end*” is a user-friendly and easy-to-understand internet-based tool that the clinician uses to input patient information, which subsequently feeds into the repository to generate assisted diagnosis and management outcomes. The user login page was succeeded by a page with patient information (patient ID, age, and ability to work/smoking/drinking alcohol as yes/no answers at the top half of the screen; occupation, ear operations, non-ear operations, medications, family history, recreational drugs with list of options for each category at the lower half of the screen). The next DSS page required the clinician to insert specific symptoms information, including vertigo/instability onset, frequency, duration and symptom free intervals; associated symptoms and preceding events (see Fig. 1A). Hovering over some of the symptom terms (e.g. oscillopsia) would provide a definition as per the Bárány Society guidelines [32].
- The clinician was then asked to insert information regarding general symptoms not associated with the vertigo/instability attacks. These symptoms were chosen by the EMBalance consortium as typically reported by patients with vertigo and dizziness and as symptoms that can help to profile the patients (see Fig. 1B). This section aimed to guide the primary care physicians to collect a comprehensive anamnesis and to collect data for the algorithm-based software driving the DSS for the final output. The physician was subsequently guided to perform a physical neuro-otological examination of eye movements, head thrust test, Hallpike positional test and gait and stance tests(see Fig. 1C, D, E), cranial nerves, blood pressure and to categorise spontaneous nystagmus if present. Each physical exam had an instructive video that could be watched by the physician prior to performing the evaluation and/or written instructions. These simple exams are relatively easy and quick to performed without expensive tools or specific equipment but may not be performed by primary care physicians.
- The DSS algorithm recommendation tool would then provide the high medium and low probability list of diagnosis, with more than one diagnosis for each patient that the physician can choose from. It would employ several data mining models for each of the diagnoses. The physician could choose one or more diagnosis from a closed set of diagnosis (see Table 2) and/or provide their own. Each diagnosis would correspond to a recommended management list (medications, vestibular rehabilitation, other) that the physician could choose and implement or ignore. A further referral option was also offered with a letter template.

**Table 2:** Non-specialist + DSS group diagnostic accuracy measures (proportions in brackets) per diagnostic categories

Diagnostic category	Sensitivity	Specificity	PPV	NPV
BPPV	73% (19/26)	89%(66/74)	70.3% (19/27)	90% (66/73)
PVD	31.4% (11/35)	92% (60/65)	64.7% (11/17)	72.3% (60/83)
BVF	50% (2/4)	98.9% (95/96)	66.6% (2/3)	97.9% (95/97)
VM	29.6% (8/27)	97.2% (71/73)	72.7% (8/11)	79.7% (71/89)
MD	100%(5/5)	96.8%(92/95)	62.6% (5/8)	100%(92/92)
Pontine/cerebellar lesion	83.3% (5/6)	90.3% (84/93)	35.7% (5/14)	97.6% (84/86)
PPPD	50% (2/4)	82.3% (79/96)	22% (2/9)	97.5% (79/81)
Cumulative measures	48.5% (52/107)	92.3% (547/592)	58.4% (52/89)	91% (547/601)

*MD* Meniere’s disease, *BPPV* benign paroxysmal positional vertigo, *PPPD* persistent postural perceptual dizziness, *BVF* bilateral vestibular failure, *PVD* peripheral vascular disease, *VM* vestibular migraine

### Outcomes measures

#### Primary outcomes

The diagnostic accuracy was measured as follows:

- Overall agreement between the diagnosis established by the non-specialist doctors (+ DSS and − DSS) and the “gold standard” as determined by an expert specialised in neuro-otology and in accordance with published evidence-based guidelines.

#### Secondary outcomes

Useability of the DSS was assessed as follows:

- Comparison of the primary care clinical diagnosis of the + DSS versus the − DSS group: sensitivity, specificity, positive and negative predictive values (odds ratio) for overall diagnosis (all diagnostic categories grouped together).
- Diagnostic accuracy of the DSS as standalone tool: comparison of sensitivity, specificity, positive and negative predictive values of the various diagnoses proposed by the DSS with high and medium level of certainty and those values in the − DSS group.
- Comparison of level of agreement between the non-specialist + DSS overall management against the “gold standard” management made by the specialist
- Comparison of level of agreement between DSS standalone tool overall management against the “gold standard” management made by the specialist
- Comparison of the number of referrals to secondary care for management in + DSS and − DSS groups.

### Sequence generation, randomisation, allocation concealment and blinding

Randomisation sequences were independently generated for each centre by the Research fellows (who were not involved with patient diagnosis or management) using Research Randomizer v4.0 software. Eligible patients were randomised in a 1:1 ratio (100 participants in each group). In accordance with the random allocation sequence, a note containing the allocation group was placed inside an opaque and sealed envelope given to the non-specialist doctor at time of recruitment. The allocation sequence was concealed from the researcher by enrolling participants in sequentially numbered envelopes. A patient’s identification trial number was assigned to each envelope to allow retrospective monitoring of patients’ allocation.

### Study procedure

**Fig. 2:**
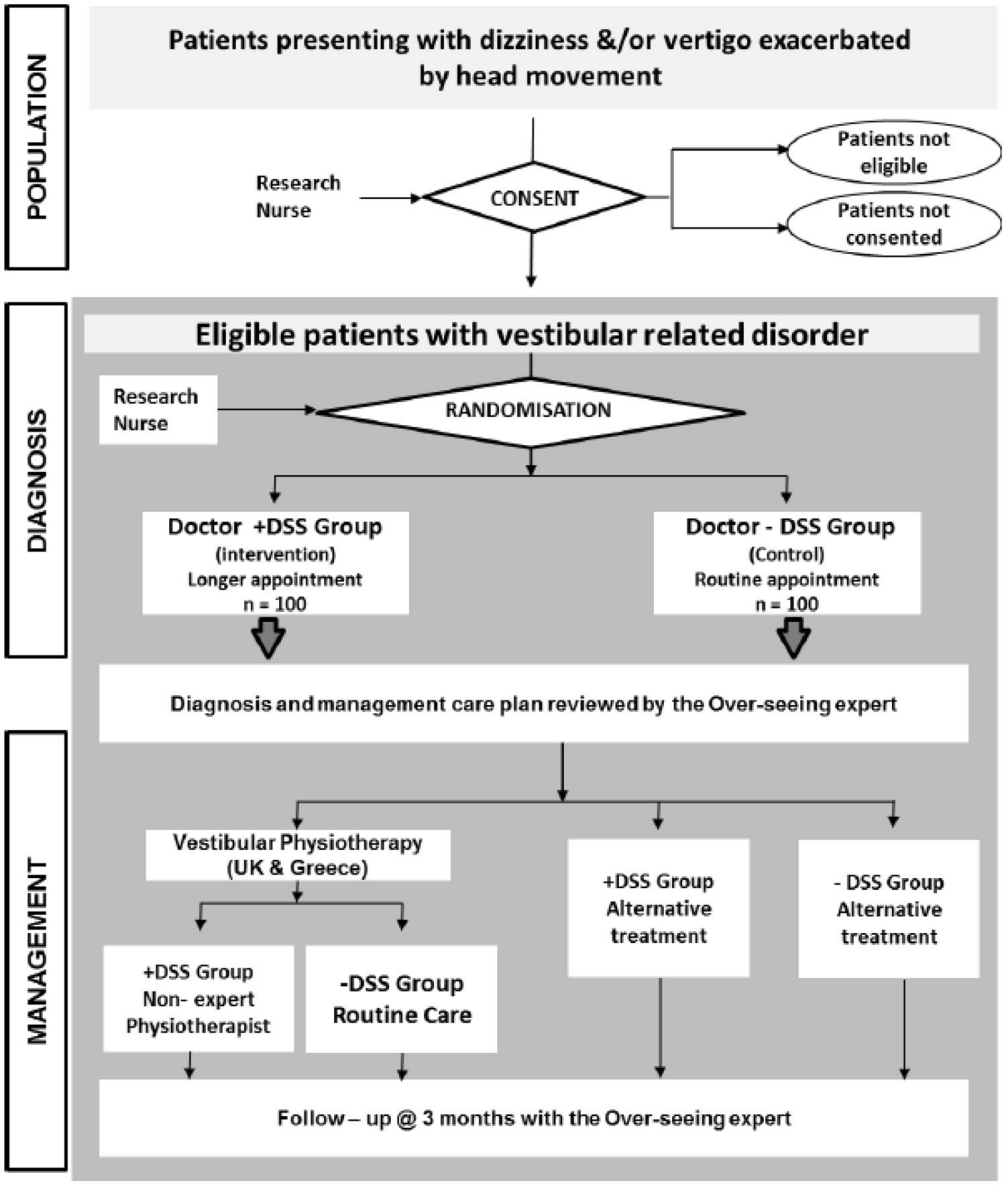
The flowchart depicts the patient progression through the phases of the EMBalance study, from screening to follow-up

The study flow is shown in Fig. 2. The clinical research nurses were responsible for patient recruitment and patient consent. They also provided to the patient their random allocation sequence and study ID number in a sealed sequentially numbered envelope that was opened by the nurse after consent.

On the day of recruitment, patients were examined by the non-specialist doctor, either with or without the DSS, according to the randomisation result. The non-specialists determined a diagnosis with and without the DSS and devised a care plan dependent on their diagnostic decision, with and without the DSS support.

The non-specialist doctors who used the DSS were told that the DSS would support them in collecting the required information for the diagnostic task, and that the EMBalance DSS platform was likely to propose more than one possible diagnosis (with probability estimation for each, ranked high, medium or low) at the end of this process, and/or suggest referral to a specialist or additional specialist investigation (e.g. MRI). The non-specialist doctors were asked to exercise their clinical judgement and to either choose one or more provided diagnosis or discard these and provide their own.

Non-specialist doctors were then asked to prescribe a treatment plan for each participant based on their diagnostic evaluation, but they also had the option to not propose a management plan and refer the patient to a specialist care centre instead. For patients allocated to the + DSS group, the non-specialist doctors were asked to either adopt the management plan proposed by the DSS, or reject this and propose an alternative treatment. The DSS proposed a management plan including pharmacological treatment, dietary intervention and/or vestibular physiotherapy. The patient was then invited to attend a specialist Neuro-otology clinic within seven days to see the supervising expert. The expert provided the final “gold standard” diagnosis, after conducting a full audio-vestibular battery of tests/other assessments as necessary, and “gold standard” management plan appropriate to the diagnosis. The “gold standard” diagnosis and management provided by the specialists in the participating centers followed the Bárány Society recommendations and a number of systematic reviews conducted by the study authors for the purposes of this project. The supervising experts then compared their “gold standard” diagnosis and management to the diagnosis and management plan provided by the non-specialist doctor, blinded in terms of whether the patient was examined by the non-experts with or without the DSS use. Both the diagnosis and management plan were deemed ‘correct’ or ‘incorrect’ based on the judgement of the experts, and in accordance with the Bárány Society guidelines. In the event that the non-specialist physicians’ decisions differed from the conclusion of the expert, the latter decided on the final diagnosis and management plan of the patient according to current evidence-based guidelines. Management included vestibular physiotherapy exercises that consisted of specialised vestibular physiotherapy (defined as personalised and supervised physiotherapy input) that was available within the clinical settings in the UK and Greece, or non- expert physiotherapy available in the other settings, that consisted of a generic booklet of Cawthorne–Cooksey exercises with instructions [33].

All patients were reviewed after 3-months in a follow-up appointment by the supervising expert independent of the management plan that was applied.

### Statistical analysis

Data analysis was performed using IBM SPSS Statistics v22.0 and StatXact statistical software package. Descriptive statistics were calculated and presented. Odds ratio(OR) and confidence intervals (CI) were calculated for the agreement between specialist diagnosis and the non-specialist overall diagnosis, the agreement between the specialist and non-specialist overall management plan in the + DSS vs. the − DSS group (primary outcomes) and rate of referrals in the + DSS vs. the − DSS group (secondary outcome). Odds ratio and confidence intervals were also calculated for onward referrals in the + DSS and − DSS. For other secondary outcomes, we calculated: sensitivity (the proportion of patients with a vestibular diagnosis correctly identified by the non-specialist with/without the DSS or by the DSS as standalone tool); specificity (the proportion of patients without a vestibular diagnosis correctly identified); positive predictive value (PPV) [the probability that participants with a provided diagnosis truly have the disease, defined as the ratio of correct diagnoses per diagnostic entity divided by the sum of this number (true positive) plus the number of suggested diagnoses not accepted by the expert (false positive diagnoses)]; and negative predictive value (NPV) [the probability that subjects without a vestibular diagnosis do not have the disease, defined as the ratio of correct negative diagnoses per diagnostic entity divided by the sum of this number (true negative) plus the number of suggested negative diagnoses not accepted by the expert (false negative diagnoses)].

## Results

In total, 200 participants were recruited and randomly allocated to the + DSS and − DSS groups. Six cases were excluded from the analysis; five patients did not attend the specialist evaluation appointment, and one patient withdrew from the study. This left 100 cases assigned to the + DSS sample, i.e. patients seen by the non-specialist doctors with the support of the DSS and 94 to the non-DSS or control group. The experts reviewed all 194 subjects, blinded to the non-specialist doctors’ final decision regarding diagnosis and management. The age range of the total group was 25–85 years (mean = 57.7, SD = 16.7) and was not significantly different in the two groups (*p* = 0.53). Of recruited participants, 37% were male and 63% female.

### Primary outcomes: diagnostic accuracy of the EMBalance DSS when used as a support tool

The non-specialist proposed diagnosis (all diagnostic categories grouped together, i.e. cumulative) agreed with the expert proposed diagnosis in 54% (*N* = 54) of cases in the + DSS use group compared to 41.5% (*N* = 39) of cases in the − DSS use group (see Tables 2, 3), odds ratio 1.35, 95% confidence intervals 0.76–2.42.

**Table 3:** Non-specialist − DSS group diagnostic accuracy measures (proportions in brackets) per diagnostic categories

Diagnostic category	Sensitivity	Specificity	PPV	NPV
BPPV	58.3% (14/24)	90%(63/70)	66.6% (14/21)	86.3% (63/73)
PVD	44.4% (8/18)	88.1% (67/76)	47% (8/17)	87% (67/77)
BVF	100% (1/1)	100% (93/93)	100% (1/1)	100% (93/93)
VM	35% (6/17)	93.5% (72/77)	54.5% (6/11)	86.7% (72/83)
MD	33.3%(2/6)	90.9%(80/88)	20% (2/10)	95%(80/84)
Pontine/cerebellar lesion	50% (5/10)	96.4% (81/84)	62.5% (5/8)	94% (81/86)
PPPD	7.6% (1/13)	95% (77/81)	20% (1/5)	97.4% (77/79)
Cumulative measures	41.5% (37/89)	93.6% (533/569)	50.6% (37/73)	92.6%(533/575)

*MD* Meniere’s disease, *BPPV* benign paroxysmal positional vertigo, *PPPD* persistent postural perceptual dizziness, *BVF* bilateral vestibular failure, *PVD* peripheral vascular disease, *VM* vestibular migraine.

### Secondary outcome measure: sensitivity, specificity, positive and negative predictive values in the + DSS and − DSS group

Sensitivity, specificity, PPV and NPV values per diagnostic category for the non-specialist + DSS group is presented in Table 2 and for the non-specialist − DSS group in Table 3. The − DSS diagnostic sensitivity was under 60% for six (out of seven) diagnoses. The + DSS diagnostic sensitivity exceeded 70% sensitivity for Menière’s disease (100%) benign paroxysmal positional vertigo (72%), and pontine/cerebellar lesions (83.3%). The DSS standalone tool diagnostic sensitivity exceeded 70% for five diagnoses.

### Secondary outcome measure: diagnostic accuracy of the DSS as a standalone tool

The diagnostic accuracy measures for the DSS proposed 1st line diagnosis (high level of certainty) and the DSS proposed 2nd line diagnosis (medium level of certainty) are given in Table 4. The sensitivity for all diagnostic categories grouped together (cumulative) was 62% with odds ratio of 3, confidence intervals 1.67–5.53 (Please note that these DSS suggestions were not necessarily adopted by the non-experts).

**Table 4:** DSS 1st and 2nd line diagnosis diagnostic accuracy measures (proportions in brackets) per diagnostic categories

Diagnostic category	Sensitivity	Specificity	PPV	NPV
BPPV	80% (20/26)	68.9% (51/74)	47.6% (20/42)	87.9% (51/58)
PVD	51.4% (18/35)	92.3% (60/65)	85.7% (18/21)	75.9% (60/79)
BVF	75% (3/4)	91.6% (88/96)	25% (3/12)	100% 88/88
VM	44% (12/27)	90.4% (66/73)	60% (12/20)	82.5% 66/80
MD	100% (5/5)	75.7% (72/95)	17.8% (5/28)	100% (72/72)
Cerebellar/pontine lesion	86% (6/7)	61.2% (57/93)	14.6% (6/41)	96.6% (57/59)
PPPD	75% (3/4)	85.4% (80/96)	15.7% (3/19)	98.7% (80/81)
Cumulative	62% 67/108	80% (474/592)	80.7% (67/183)	83% (474/517)

*MD* Meniere’s disease, *BPPV* benign paroxysmal positional vertigo, *PPPD* persistent postural perceptual dizziness, *BVF* bilateral vestibular failure, *PVD* peripheral vascular disease, *VM* vestibular migraine.

For the cumulative results of number of cases correctly and incorrectly diagnosed by the DSS across the four clinical sites across centres, there was agreement between the DSS proposed 1st line diagnosis (high level of certainty) and expert diagnosis in 42 (42%), and with the DSS proposed 2nd (medium level of certainty) diagnosis in another 21 (21%) of cases, with no agreement between DSS proposed 1st and 2nd level diagnosis with expert diagnosis in 37 (37%). Number of cases with agreement between the DSS first line diagnosis and the expert versus agreement between the DSS second line diagnosis and the expert were merged, to assess diagnostic accuracy of the DSS for first- and second-line correct diagnosis taken together. Agreement with expert diagnosis was thus observed in 63% of cases and significantly better than no agreement with expert diagnosis observed in 37% of cases at *p* = 0.009.

The difference between correct cumulative DSS proposed diagnosis and the correct diagnosis by non-experts without DSS use was statistically significant at *p* value of 0.0039.

### Secondary outcome: management agreement (+ DSS to gold standard, DSS standalone) and number of referrals to secondary care in + DSS and − DSS groups

**Fig. 3:**
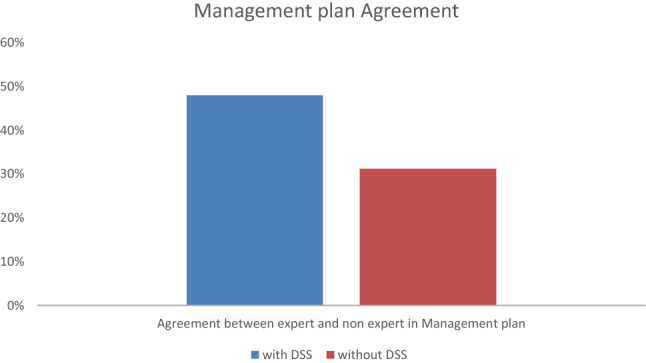
Agreement percentage between specialists and non-specialists with and without DSS in management plan

Correct management by the non-specialists (i.e. on the basis of agreement with expert management) was observed in 48% (*N* = 48) of cases in the + DSS vs. in 31% (*N* = 29) of cases in the − DSS group (Fig. 3), odds ratio 2.07, 95% confidence intervals 1.15–3.72.

In the cases in which the diagnosis proposed by the non-expert was correct (54 out of 100), within the + DSS group, management proposed by the DSS was correct in 37 cases and incorrect in 12 cases (five cases were missing management), while in those with a correct diagnosis in the − DSS group (*N* = 39 with correct diagnosis, one case missing management), management was correct in 25 and incorrect in 13 cases, OR 0.6237, CI 0.2450–1.5878.

**Fig. 4:**
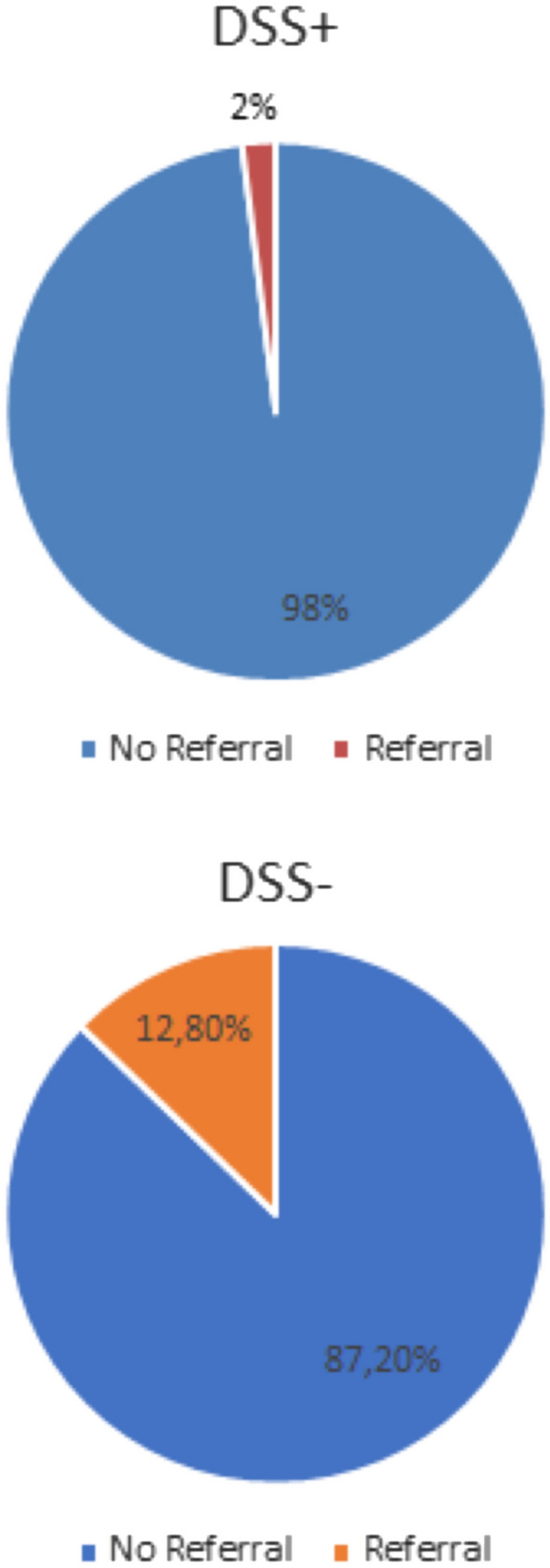
Patient referral (percentage) for management in + DSS and − DSS group. *NR* no referral, *R* referral

There was a significantly higher proportion of participants referred from the − DSS (12.8%) onwards to specialist services for additional evaluation of their symptoms compared to the + DSS group (2%) (Fig. 4), odds ratio 7.17, 95% confidence intervals 1.56–32.96.

## Discussion

This is the first completed clinical study on the use of a novel DSS for diagnosing vestibular disorders in a primary care setting. We found positive trends for improved diagnosis as well as management in the + DSS compared to the − DSS group and a better diagnostic and management accuracy for the standalone DSS than the + DSS group.

Management of dizziness depends on the underlying cause. However, dizziness complaints of patients presenting to primary care are heterogeneous [34] and diagnosing the dizzy patient can be challenging in the presence of a limited diagnostic strategy [35]. Previously, machine learning algorithms and predictive models have been used in tertiary healthcare systems [28, 36–38]. One key feature of the EMBalance diagnostic decision support system (DSS) was that it aimed to address symptom definition and other diagnostic strategy barriers towards improved diagnostic success. The initial EMBalance validation study yielded promising results.

### DSS as a support tool

The diagnostic evaluation of non-specialist physicians as judged against the specialist’s diagnosis tended to be better in the + DSS group compared to − DSS (54% vs. 41.5% correct in the two groups, respectively). When all diagnostic categories were considered together, there was also a weak trend for the + DSS group to have a better sensitivity and PPV than the − DSS group (see Tables 3, 4). The possible reason for this weak trend could be that the non-specialists did not always opt to adopt the DSS high and moderate certainty proposed diagnosis. Another reason could be the utilisation of unique specialist language in the DSS, which the primary physician was unfamiliar with. A recent study showed that dizzy patient characteristics are semantically represented by specific language patterns, and such representation associates strongly with specific vestibular conditions [37]. Patients often use the term ‘dizziness’ to describe their symptoms. With a varied differential diagnosis and lack of knowledge in vestibular disorders among the primary care non-specialists, it can be challenging for the clinicians to acquire relevant information to make a correct diagnosis, even with the support of a DSS. The front end graphical user interface is both user-friendly and easy-to-understand, according to user feedback (that was conducted as a separate study within the EMBalance project). Programme language understanding was thus not required, and it was not identified by the users as an issue. The DSS backend included clinical data collected from 984 patients’ records, with patients characterised by approximately 350 features. It is indeed possible that a bigger number of cases and/or additional features would increase the likelihood of accurate diagnosis. It is also possible that history and clinical examination were not conducted properly by non-expert clinicians. While the EMBalance offered an additional toolbox with a series of instructional videos on how clinical examination should be correctly conducted and reported, available in the interface and as a link in the relevant youtube channel (https://www.youtube.com/channel/UCXFf98Ktus48Ut9a5Nbm4sA), physicians were not explicitly instructed to watch these before DSS use. These factors will be addressed in the next iteration of the DSS.

### DSS as a standalone tool

The DSS standalone tool provided 1st and 2nd line diagnostic decisions and had a better sensitivity than that of the − DSS non-specialist group (Odds Ratio of 3), although inevitably its specificity was weakened. The overall sensitivity of the DSS was 62%, similar to a study by Feil et al. [28]. The sensitivity of the EMBalance DSS standalone tool was high (> 80%) for MD, cerebellar pontine lesion, and BPPV diagnoses, and medium (> 70%) for PPPD and BVF diagnoses, but relatively low (< 50%) for VM and PVD. Feil and colleagues also reported low sensitivity in VM diagnosis, attributing this to the fact that vestibular migraine is a diagnosis of exclusion. The DSS could not entirely replace clinical expertise, which makes clinical reasoning, i.e. hypothesis driven focussed clinical information gathering, more efficient [39]. In future, some of these issues may be answered by assessing how non-specialist expertise level affect diagnostic outcome, or whether within expert increased experience after a period of DSS usage may improve diagnosis.

### Individual diagnostic entities

For primary care practitioners including general practitioners (GPs), key priorities for their clinical practice are to exclude a life-threatening disease, or diagnose a treatable specific disease, and to identify a chronic development of dizziness to stop evolution of dizziness into a chronic condition [34]. A further analysis based on the DSS capability to propose a correct diagnosis, on the basis of individual diagnostic entities, showed that the EMBalance platform had high sensitivity and reasonable specificity for some high clinical impact diagnoses. This was observed for cerebellar-pontine lesions that can be life-threatening and require early diagnosis [12, 13]. The sensitivity of the non-specialist physicians − DSS diagnosis of cerebellar-pontine lesions was only 50%. The sensitivity of the EMBalance DSS system to diagnose cerebellar-pontine lesions was higher than the iPad-based medical device used in Feil’s study [28], possibly due to the inclusion of highly relevant clinical history and examination as per the TiTrate and HINTS rules [16] in conjunction with the applied data mining techniques [27]. The sensitivity of the DSS was also high for common vestibular disorders such as BBPV, or PPPD that are effectively treatable once identified [40, 41], and for rarer disorders such as MD. To this end, the DSS holds promise for the diagnosis of theses vestibular conditions, as indicated by the results of this randomised clinical trial.

### DSS management plan

Management was significantly better in the + DSS vs. in the − DSS group (odds ratio 2.07). This is in agreement with the findings of a meta-analysis of 138 (non-vestibular) DSSs clinical trials that reported improved quality of treatment prescribed by the clinicians in 46 of these studies [42].

The management results suggest that despite the large proportion of patients presenting with dizziness and vertigo in general practice, non-specialist physicians’ prescription of vestibular management options remains sub-optimal. The proportion of participants who were referred for further assessment and management was also significantly lower in the + DSS group (2%) vs. the − DSS group (12.7%). Referral to expert recommendation and treatment is one of the key outcome measures that judges success of DSSs [42]. The mean number for patient visits to their Health Care providers required to establish a correct diagnosis and start appropriate treatment, both in the US and the UK, is 4.5 [8], so this improvement in correct management and reduction in referrals would be significant in terms of actual costs to the Health Systems and Society. Management decision accuracy was also high for the DSS at 75%. Issues relating to mistrust of newly developed technological solutions [43] could also impact on take up of the DSS management recommendations, since non-specialists management decision was correct in 56% and was lower than the 75% rate of the DSS. Another aspect to consider is that clinicians perceive the use of a decision support system as more advantageous after using the system for a while, as opposed to at the start of usage [44]. Again, looking into change of non-expert management decision making at the start of DSS use vs at the end of DSS usage may offer some insights into user adoptability.

Overall, just under 30% of the EMBalance study participants were given appropriate management by the non-specialists − DSS, despite the moderate to strong evidence basis for the effectiveness of some low-tech interventions for dizziness, such as vestibular rehabilitation [45]. Optimal management of dizziness differs significantly according to the underlying vestibular condition [46], however, it is possible for most patients to be managed within the primary care setting [47]. Trained family practice staff have limited confidence in treating vestibular disorders [48, 49], while specialist health professionals such as audiologists are highly qualified to perform vestibular assessment, but they are underprepared by their graduate training [50] to undertake management. Not surprisingly, the most widely accepted primary care practice for patients requiring vestibular rehabilitation consists of patient’s self-management of symptoms with support of a validated booklet-based VR programme (available, for example, from the Ménière’s Society UK [33]. And while this generic form of management leads to significant patient improvement [33], this improvement is considerably less than multidisciplinary, individualised management [51] that can be supported to some extent by the EMBalance DSS.

Our results indicate that vestibular management is generally unavailable to patients who are managed solely at primary care level or by non-specialists. This is a significant issue since these patients are three times more likely to develop psychological sequelae such as anxiety, panic disorder and depression [52], and chronicity of dizziness [53], resulting in a high socioeconomic cost [54]. These patients should receive early diagnosis and appropriate management. The EMBalance DSS holds promise in this respect. However, diagnostic accuracy of the current DSS would need to be improved before it is adopted in for clinical use, while the results will need to be replicated in a larger multicentre trial.

### Limitations and future research

The use of a computer-aided system may to some extent disrupt the patient–doctor relationship [21]. The design of the DSS interface, ease in entering patient data and increased appointment time given to consultation were some preliminary measures adopted to reduce this limitation and the patient discomfort. Furthermore, a special section in the users’ manual provided to participating doctors was dedicated to informing users regarding this issue and suggesting strategies to reduce this risk.

The lack of confidence in information technology solutions, even when these are well validated, is a well reported issue that needs to be considered when implementing such technologies [43]. Adoptability may require educational courses and a strong customer acquisition to be able to fully exploit the potential of these new technologies. Another limiting factor that may have influenced diagnostic accuracy results for both the non-specialist and the DSS would be whether non-specialist physicians’ inexperience may affect their ability to correctly elicit clinical information, for example by phrasing appropriate questions on key symptoms suggested by the DSS, ultimately biasing the capability of the DSS to predict the correct diagnosis [37]. A further challenge for the non-specialist is the existence of multiple vestibular disorders, for example the overlap of vestibular migraine with Meniere’s [55]. These data would need to first inform and guide subsequent DSS iterations and then be replicated by additional studies. The study did not log the percentage of missing data in the fields of the EMBalance DSS that were populated by the clinicians, to assess how this could also influence the diagnostic outcome. This would be worth exploring in bigger studies than the current to investigate optimal number of populated features required for accurate diagnosis. Finally, the Covid-19 pandemic has highlighted the need for remote diagnostic tools, and the potential value of detailed, personalised and digitally recorded data towards precision healthcare [56], and a modified version of the EMBalance tool would potentially be well suited and should be explored for this purpose.

The next version of the EMBalance DSS will incorporate some measures to improve the DSS performance in terms of diagnostic accuracy. The training data in the back-end, on which the DSS prototype was trained per diagnostic category, will be increased. The DSS will incorporate additional questions for review of important features that do not match (e.g. reported vertigo with duration of hours with a positive Dix-Hallpike test). It will also include specific rules that could highlight important features from the history and clinical examination (eg as per the Consensus on Virtual Management of Vestibular Disorders) [25], so that the next version will be a “hybrid” system, based both on rules as well as data mining techniques to enhance the predictive ability of the system. Finally, as per recommendations by target users’ feedback at the end of the study, and similar to the more recent PoiSe study [29] it will include an introductory course for the target users, to explain the DSS structure and how data population should be conducted.

## Conclusion

The EMBalance DSS provides a structured and detailed diagnostic and management plan for a comprehensive list of vestibular disorders. The diagnosis and treatment plan available through the system has been developed with the input from the EMBalance consortium, in accordance with national and international guidelines. This proof-of-concept study showed a trend for improved diagnosis of vestibular patients with the use of the DSS vs. without that was statistically significant when the first- and second-line diagnosis were accepted by the primary care physician, and in the provision of a significantly better management strategy. Implementation of a decision support system such as the EMBalance DSS for simple to more complex cases (where none or limited improvement is shown after three months follow up), may improve patients’ diagnosis and symptoms with a direct positive effect on the associated socio-economic costs, and patients’ quality of life. The EMBalance DSS will require further development to improve its diagnostic accuracy, but holds promise in ensuring that patients with a vestibular disorder are diagnosed and managed in a timely and effective manner, and may be of particular relevance in the Covid-19 pandemic era. New interactive ways to communicate with patients have emerged from the recent Covid-19 pandemic, and we believe that in the future DSS and AI strategies like the EMBalance may become a concrete reality to improve patient care.

## Data Availability

The data that support the findings of this study are available upon request from the University College London, but restrictions apply to the availability of these data. The data underlying this article cannot be shared publicly.

## Abbreviations

BPPV: Benign paroxysmal positional vertigo
BVF: Bilateral vestibular failure
DSS: Decision support system
− DSS: Control group: ‘non-specialist doctors without the support of the DSS’
+ DSS: Intervention group: ‘non-specialist doctors with support from the DSS’
MD: Meniere disease
MHRA: Medicines and Healthcare products Regulatory Agency
MV: Vestibular migraine
PPPD: Persistent postural perceptual dizziness
PVD: Peripheral vestibular disorder
RCT: Randomised control trial
